# Concomitant Campylobacteriosis in a Puppy and in Its Caregiver: A One Health Perspective Paradigm in Human-Pet Relationship

**DOI:** 10.3390/vetsci10040244

**Published:** 2023-03-24

**Authors:** Alessia Candellone, Paola Badino, Flavia Girolami, Matteo Cerquetella, Patrizia Nebbia, Luca Aresu, Simona Zoppi, Domenico Bergero, Rosangela Odore

**Affiliations:** 1 Department of Veterinary Sciences, University of Turin, 10095 Grugliasco, Italy; 2 NutriTO Vet srl., 10090 Rosta, Italy; 3 School of Biosciences and Veterinary Medicine, University of Camerino, 62032 Camerino, Italy; 4 Istituto Zooprofilattico Sperimentale del Piemonte, Liguria e Valle d’Aosta, 10154 Turin, Italy

**Keywords:** One Health approach, raw diet, *Campylobacteriosis*, zoonoses, puppy

## Abstract

**Simple Summary:**

Dogs are considered family members and a growing amount of attention has been dedicated to their nutrition by pet parents during last decades. The administration of unbalanced, poultry-based raw diets (PRD) has represented a rising trend, with some nutritional and microbiological implications on both pets and human wellbeing. Here, we report a case of a laboratory-confirmed *Campylobacter (C). jejuni* and *C. upsaliensis* symptomatic infection in a puppy, a French Bouledogue, female, 6 months of age, and fed a PRD, and in its owner. Both recovered after appropriate diagnostic investigations and treatments: however, hospitalization and a long course of antimicrobials were necessary to fight the multi-drug-resistant infection. This case underlines the potential risk of *Campylobacter* spp. exposure related to current pet food fads, highlighting the importance of the One Health approach, where veterinarians, physicians and caregivers need to develop effective strategies for the prevention of zoonoses spread.

**Abstract:**

We report a case of laboratory-confirmed *Campylobacter (C). jejuni* and *C. upsaliensis* symptomatic infection in a puppy, a French Bouledogue, female, 6 months of age, fed a raw, unbalanced, poultry-based diet (RPD), (48.1 CP, 33% EE, 0.3% Ca, 0.5% Phos, 0.5 Ca/P, on a dry-matter basis), and in its owner. Soon after adoption, the pet and the caregiver showed severe gastrointestinal signs and needed hospitalization. Fecal PCR assays, selective cultures, and antibiotic susceptibility testing were performed, and multi-drug resistant *C. jejuni* and *C. upsaliensis* were isolated from the feces of both. The same bacteria were also identified by FISH in the dog colonic biopsies collected during endoscopy. The puppy was prescribed a complete commercial diet for growing dogs, (30.00% CP, 21.00% EE, 1.2% Ca; 1% Phos; as fed) and treated with ciprofloxacin. The dog and the man healed uneventfully and tested negative for further fecal PCR analyses. This report focuses on dog nutritional management and explores the potential routes of exposure, with emphasis on emerging outbreaks related to current pet food fads. Our data support the One Health approach, where veterinarians, physicians, and owners are challenged to build effective stewardship to prevent the spread of zoonoses.

## 1. Introduction

In humans, campylobacteriosis is considered a leading cause of bacterial gastroenteritis worldwide [1,2,3]. A *Campylobacter* spp. surveillance study, performed in Italy on human stool samples from 2013 to 2016, showed that *Campylobacter (C.) jejuni* was the most prevalent species (73.4%), with a high rate of antimicrobial resistance [1]. Food is the primary source of human Campylobacteriosis, but the human–animal boundary represents a risk factor for the bidirectional transmission of this infection, and dogs can play a role as a reservoir [2,3,4]. In recent years, the administration to pets of home-made or commercial raw-meat diets has become markedly more popular among pet parents in many developed countries, including Italy, due to consumer perception of providing a more “natural” feeding [5,6,7,8,9,10]. A large, structured survey performed in the USA by the American Pet Products Association indicated that 17% of dog owners administered their pets with raw or cooked human food, and 3% of dogs and 4% of cat caregivers were reported purchasing raw pet food at the market [11]. Due to this rampant habit, a series of food-borne disease outbreaks have been reported in the literature, with an increased risk for *Campylobacter jejuni* and *Campylobacter coli* infection of pet origin in dog owners [3,4,12,13].

Moreover, according to Davies et al. [14], the prevalence in dogs of *Campylobacter* species, *C. jejuni* and *C. upsaliensis* was 36%, 13% and 23%, respectively. *Campylobacter* species were isolated from 28% (*C. jejuni* from 22%) of retail raw-meat petfood samples, and poultry meat was more likely to be positive than non-poultry meat [14]. In another recent study, *Campylobacter jejuni* and other bacteria, such as *Salmonella* sp. and *Listeria monocytogenes*, were isolated from 33.3% fecal samples collected from dogs fed a raw-meat diet [15]

Here, we report a case of laboratory-confirmed *Campylobacter jejuni* and *C. upsaliensis* concomitant symptomatic infection in a puppy, fed a poultry-based raw diet (PRD) and in its caregiver.

## 2. Case Presentation

### 2.1. Signalment, Anamnesis and Clinical Findings

A 6-month-old, female, French Bouledogue, weighing 5.8 kg, was admitted to the veterinary hospital for worsening entero-colic signs. The main complaints were bloody diarrhea (hemorrhagic colitis) and progressive lethargy, which had been occurring for three days. Symptomatic treatments (i.e., probiotics, adsorbents and oral fluid therapy) prescribed by the general practitioner seemed unhelpful in reducing the dog’s clinical symptoms, which worsened to the point that hospitalization was needed.

The dog was adopted at the age of 2 months from a kennel club of recognized reputation; it was regularly dewormed and vaccinated and was fed, since its arrival in the new household, a PRD, self-formulated by the owner without professional consultation.

In the previous weeks, the puppy had already been showing scattered gastro-intestinal signs associated with stunted growth, but those symptoms have been minimized by the caregiver, who used to associate them with dietary indiscretion. At the time of hospital admission, the patient’s presentation seemed more severe, and the management of the case was made even more difficult by the fact that the owner himself had to be admitted to the local hospital due to the appearance of overlapping symptoms.

**Figure 1:**
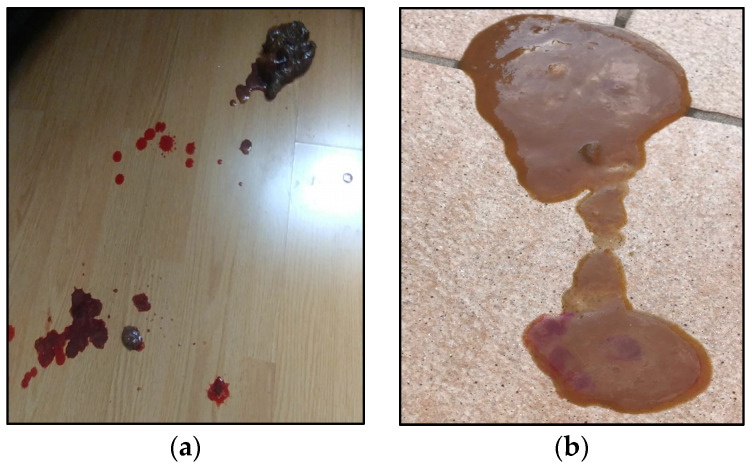
Puppy’s fecal characteristics upon presentation (**a**,**b**) the day before hospitalization.

During the emergency room (ER) consultation the dog was quiet, lethargic, hyperthermic, mildly dehydrated, and showed abdominal discomfort and a fecal score of 5 out of 5 [16] (Figure 1).

### 2.2. Differential and Diagnostic Work-Up

The dog was first hemodynamically stabilized in the Intensive Care Unit. Then, the author (A.C.) performed a complete diagnostic workup, taking into consideration that acute and severe colitis in such a young patient could be non-self-limiting and potentially life-threatening. The main clinical and biochemical findings recorded during ER examination are synthesized in Table 1.

Endoparasites, dietary indiscretion, bacterial enterocolitis, hemorrhagic gastroenteritis syndrome and extra gastrointestinal-tract diseases, such as hypoadrenocorticism, represented our main differentials. Fecal flotation, exfoliative rectal cytology, laboratory analysis including a complete hematobiochemical profile, basal cortisol and Parvo SNAP test (IDEXX) were then performed. Moreover, survey abdominal radiographs were also performed to seek out “sentinel loops”, as a possible consequence of ileocolic intussusception or foreign bodies ingestion.

Both the fecal analysis and the Parvo SNAP test had negative results, and fecal cytology did not show any sporulated organisms compatible with *Clostridium perfringens* or bacteria with the typical curved rod appearance of *Campylobacter* sp. Hematobiochemical abnormalities, represented by mild leukocytosis, hypocalcemia, hyponatremia and increased BUN and ALT serum concentrations, were considered nonspecific and compatible with alteration described in patients with acute enteropathies of unknown origin [18,19]. An Addisonian crisis was also ruled out being the basal cortisol >2 µg/dL [20]. A summary of laboratory findings recorded during the diagnostic workup is depicted in Table 2. Abdominal radiograms revealed diffuse large-bowel dilatation with gas/fluid levels; no foreign bodies were detected.

Given the above, bacterial enteritis and hemorrhagic gastroenteritis syndrome represented the most likely diagnosis. To further address the case, a puppy nutritional assessment and a phone discussion with the owner’s physician were scheduled.

**Figure 2:**
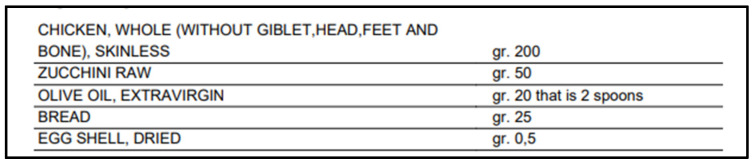
List of ingredients of the PRD. The image represents part of the PDF report provided by Myvetdiet^©^ software after appropriate elaboration (English version, automatic translation from Italian language). RMD: raw meat diet; Gr: grams.

The nutritional profile of the dietary regimen was extrapolated by using a common software for home-made diets formulation (Myvetdiet©, URL http://myvetdiet.it/app/benvenuto.asp, accessed on 1 January 2022); ingredients and detailed nutritional analysis are depicted in Figure 2 and Table 3, respectively. Briefly, 48.07% Crude Protein, 33% Ether Extract, 0.3% Calcium, 0.51% Phosphorus and 0.51 Calcium/Phosphorus ratio were estimated, resulting in a hypocaloric regimen as compared to NRC guidelines for growing puppies, with some minerals (i.e., Iron, Zinc, Copper, Selenium, Iodine, Manganese) and vitamins (i.e., B2, B12, D), the intake being lower than minimum requirements [21,22]. Moreover, the administration of raw poultry meat was identified as a possible source of food-borne pathogens, conceivably responsible for acute bacterial enteritis, such as *Salmonella* and *Campylobacter* spp. [14,15,23,24].

At the same time, the owner’s physician informed one of the authors (A.C.) that the owner was diagnosed with a multidrug-resistant *Campylobacter jejuni* infection, based on fecal selective cultures.

An impromptu “One health” task force including the owner physician and selected veterinarians (a general practitioner, F.M., a nutritionist and gastroenterologist, A.C., a pathologist, L.A. and two microbiologists, P.N. and S.Z.) was therefore established. Fecal samples from the dog and the owner were then collected and submitted to the Istituto Zooprofilattico Sperimentale of Piemonte, Liguria and Valle d’Aosta for (a) selective fecal culture and antimicrobial susceptibility testing and (b) polymerase chain reaction (PCR) assays for *Campylobacter* spp. In detail, fecal samples were analyzed according to ISO 10272-1:2017 [25]. Identification of *Campylobacter* species was performed by biochemical system API Campy (API-bioMérieux SA, Marcy l’Etoile, France) and by MALDI-TOF technology (MALDI Biotyper, Bruker Diagnostic, Bremen, Germany).

Moreover, a complete gastroenteric and respiratory tract endoscopy was performed to grade the Brachycephalic obstructive airway syndrome (BOAS). Gastric, duodenal, and colonic biopsies were also collected for histopathology and/or fluorescent in situ hybridization (FISH). Colonic biopsies were submitted for adjunctive microbiological analysis as well. The results of the analyses are summarized in Table 4, Table 5 and Table 6.

Briefly, main endoscopic abnormalities were represented by stenotic nares, a mildly elongated soft palate with a I grade laryngeal collapse, aberrant nasopharyngeal turbinates and a diffuse gastro-entero-colic inflammation, as a result of primary or secondary alterations associated with BOAS. Histopathological analyses were compatible with mild multifocal chronic gastritis and moderate-to-severe active lymphoplasmacytic enterocolitis, with signs of colon mucosa erosion. The patient fecal culture and PCR assays tested positive for *Campylobacter upsaliensis* and *Campylobacter jejuni*, respectively. *Campylobacter jejuni* infection was confirmed by colonic biopsy culture and FISH analysis on histological smears. Antimicrobial susceptibility testing revealed multi-drug resistant *C.* strains, with a common sensitivity limited to Ciprofloxacin. PFGE cluster analysis confirmed a genetic association between *Campylobacter* spp. isolates from human feces and dog fecal and colonic samples (data not shown).

### 2.3. Treatment and Follow Up

The puppy was administered with Ciprofloxacin, 10 mg/kg q12 os (Ciproxin^®^ 250 mg, tablets). The drug was used off-label, based upon antimicrobial susceptibility reports than reported that other veterinary-licensed antimicrobial drugs were ineffective in controlling the infection; and a complete extruded commercial diet for growing dogs (30.00% CP, 21.00% EE, 1.2% Ca; 1.05% Phos; as fed) was prescribed. Ingredients were represented by: fresh lamb meat 40%, meat byproducts, potato starch, peas, pea protein, linseed, lamb gravy, sunflower oil, minerals, fish oil, botanical herbs 0.2% (marigold blossoms, nettle, blackberry leaves, fennel, caraway, chamomile, balm), mannanoligosaccharides (MOS), fructo-oligosaccharides (FOS), carrots 0.04%, apples 0.04%, pears 0.04%, broccoli 0.04%, banana 0.04%, kale 0.04%, spinach 0.04%, beetroot 0.04% and blueberries 0.04%.

Daily energy requirements (DER) for growth were calculated as follows [22]:


DER (Kcal/ME/day): 210 kcal/kg × BW^0.75^

ME: metabolizable energy;

BW: Actual body weight;

An adult BW of 11 kg was estimated.

Given an energetic density of the diet of 4350 kcal ME/1000 gr, as fed, a total of 180 g was administered daily, divided into three meals. Fresh and clean water was allowed ad libitum.

A dietary supplement (Pet-Mod Intestinal F^®^, Prosol, single dose sachets of 2 g of powder each) mainly containing psyllium husks powder (450 mg), I-Care complex (hydrolized yeast from *Kluyveromices fragilis*, 400 mg) and a multi-strain probiotic (Procanicare, Ecuphar) consisting of *Lactobacillus fermentum* NCIMB 41636, *Lactobacillus plantarum* NCIMB 41638, *Lactobacillus rhamnosus* NCIMB 41640 (1a001) and 3 × 10^11^ UCF/Kg, were also prescribed for 21 days. Daily doses were selected upon producer indications.

Moreover, family members were educated to wash their hands thoroughly right after touching the dog, cleaning up excrement and handling feed and food. Further administration of raw or undercooked food was also discouraged for the future.

Both the owner and the dog recovered uneventfully and were discharged from the hospital 4 and 6 days after their admission, respectively.

One month after the diagnosis, the patient’s clinical and nutritional reassessment were performed. The dog appeared bright and alert; body weight resulted in line with expected growing curves (BW after 1 month: 7.4 kg; BCS 4.5/9) and a fecal score of 2/5 was reported by the owner. All further fecal analyses tested negative for *Campylobacter* spp. The caloric reassignment was performed to adapt the daily energetic intake to current requirements and a surgical correction of BOAS anatomical abnormalities identified during endoscopic assessment was scheduled.

## 3. Discussion

The manuscript describes the clinical presentation, diagnostic work-up, nutritional, and therapeutic management of *Campylobacter* spp. enterocolitis in a puppy. In addition, we highlight the importance of a “One-health approach” while dealing with food-borne pathogens for which pets may represent possible reservoirs. Indeed, here, both the dog and its caregiver have been diagnosed with *Campylobacter jejuni* and *Campylobacter upsaliensis* symptomatic infection, caused by genetically associated bacterial strains.

*Campylobacter* spp. is commonly found both in healthy and diarrheic dogs and cats. The presence of *Campylobacter* is hardly surprising in young and/or stressed animals, regardless of their health status. In canine patients, the catalase-negative species *Campylobacter upsaliensis* tends to dominate, accounting for up to 96% of isolates. However, the relevance of *Campylobacter upsaliensis* for animal health is still debated, and robust scientific evidence that it can be responsible for illness in pets is lacking. On the contrary, *Campylobacter jejuni,* is the most common catalase-positive species [28], and presumably the most common cause of clinical campylobacteriosis, even though it can be found in normal individuals [23]. Clinical manifestation of campylobacteriosis is non-specific and includes diarrhoea (bloody or watery), abdominal pain and fever [29]. Campylobacteriosis is most frequently a self-limiting disease; however, sometimes antimicrobial treatment is required [30]. These findings are consistent with our case study: The dog was in fact hospitalized due to acute and complicated hemorrhagic colitis caused by both *Campylobacter jejuni* and *Campylobacter upsaliensis* enteritis and needed a specific antimicrobial treatment. Although it is not possible to determine the individual contribution of each bacterial species to clinical symptoms, it is likely to assume that signalment and nutritional management had a role in the pathogenesis of the disease.

In fact, *Campylobacter* spp. virulence may be influenced by several aspects [29,30,31], and risk factors in dogs are represented by young age, stress, living conditions, season, geographic area, model of feeding and breed [30]. In detail, Andrzejewska et al. [32] reported the highest prevalence of *Campylobacter* spp. in canine patients less than one year of age. Westgarth et al. [33] observed that younger dogs were more likely to harbor *Campylobacter upsaliensis* and *Campylobacter jejuni* than adult individuals, maybe due to their lower immunological competence and immature gastrointestinal tract microbiota. Regarding nutritional habits, the emerging trend of feeding pets raw meat-based diets is considered a hazard for foodborne diseases outbreaks described in both humans and cohabitant quadrupeds [12,15,24,34]. Raw-meat dietary regimens, sometimes labeled as “Bones and Raw Food” or “Biologically Appropriate Raw Food” (BARF) diets, include uncooked ingredients from either livestock or wild animals and may be home-prepared or commercial, with the latter being supplied as freeze-dried, frozen or fresh diets. Source animal species for those feeds appear to be disparate, but poultry meat frequently represents the main meat ingredient of European formulations, likely due to the accessible cost of this prime matter [5,8,9]. The patient described here was fed a home-made unbalanced PRD regimen, based on the caregiver’s perception of providing a more “natural” and “healthy” diet. As poultry meat has been identified as a major source of food-related transmission of Campylobacter spp. to humans and pets, feeding dogs with contaminated chicken carcasses may contribute to a higher spread of infective doses of *Campylobacter* spp. into the household. Unfortunately, microbiological analysis of the PRD, of the dog fomites and of the kitchen utensils was not performed; thus, the primary source of the infection could not be clearly identified. Moreover, the nutritional evaluation of the PRD fed to the patient showed a daily caloric gap of around 300 kcal/ME and several minerals and vitamin deficiencies. These factors may have acted synergistically with the poor microbiological quality of the regimen in determining a *Campylobacter* symptomatic infection.

As stated before, antimicrobial treatment was necessary to obtain the puppy’s clinical healing. However, it must be stressed that antibiotic administration to treat acute diarrhea is usually discouraged. According to Candellone et al. [35], the proper medical management of diarrheic dogs mainly relies on nutritional and symptomatic interventions, with antimicrobial prescriptions only reserved to complicated cases or to those patients in which an etiological diagnosis has already been made. Our clinical and microbiological records provided robust evidence for a specific bacterial infection, and pharmacological treatment was selected according to its susceptibility testing. Worth noting is the multi-drug antimicrobial resistance profile of isolated *Campylobacter* strains [36,37,38,39]. In humans, macrolides remain the frontline agents for treating culture-confirmed *Campylobacter* cases [40]. Quinolones (i.e., ciprofloxacin) are also commonly administered because of their use in the empirical treatment of undiagnosed diarrheal illness, such as travelers’ diarrhea [41]. Quinolones represent the most prescribed drugs for the treatment of canine symptomatic Campylobacteriosis, as well. However, prudent use in young patients, based on antimicrobial susceptibility testing, is always mandatory.

Regarding diagnostic work-up, several tests may be helpful while dealing with a suspected campylobacteriosis, despite this infection is often underdiagnosed in the clinical setting. In fact, the identification of a *Campylobacter* enteritis is challenging, largely because of the high prevalence in healthy animals but also because of issues regarding the successful isolation of *Campylobacter* spp. [23,29,30]. Furthermore, the caregiver’s economic restraints and/or misuse of antimicrobial drugs represent limiting factors for a specific diagnosis. Fecal cytology and cultures, and molecular diagnostic techniques represent possible diagnostic tests. However, in our patient rectal cytology tested negative for the typical curved rod appearance of *Campylobacter*, maybe because of the low specificity of such an assay, or due to the paucity of fecal material collected at that moment. It is important to underline that such a test only identifies *Campylobacter*-like organisms (CLOs), not necessarily *Campylobacter* spp. Other organisms, such as *Anaerobiospirillum*, *Arcobacter* and *Helicobacter*, have a similar morphology and *Campylobacter* and CLOs are commonly present as part of the normal microflora. Furthermore, appearance does not differentiate between pathogenic and harmless commensal species. At best, the detection of CLOs is mildly suggestive of campylobacteriosis, but this test has essentially no clinical utility. Anyhow, the combination of different screening methods and the concomitant need for a gastrointestinal tract endoscopy for BOAS (PCR + fecal cultures + FISH on histological smears) allowed the authors a prompt and accurate diagnosis with intramucosal specific bacterial identification, reinforcing the hypothesis of a direct correlation between clinical presentation, endoscopic appearance, histologic lesions, and microbiological isolation.

Fortunately, both the dog and the owner healed without consequences, but several scientific references report that life-threatening or long-lasting diseases, such as chronic Inflammatory Bowel Disease (IBD), Barret’s esophagus, Guillain–Barré Syndrome and Miller–Fisher Syndrome, could occur [42]. These data reinforce the need for a proactive interaction between caregivers, veterinarians, and physicians in order to establish common preventive and diagnostic guidelines to preserve global health and assure a human–companion animal bond free of zoonotic hazards.

## 4. Conclusions

Campylobacteriosis represents an emerging zoonosis and an underdiagnosed cause of canine acute enteritis. This case report underlines the importance of the One Health approach when healthcare providers and diagnostic institutes are challenged to develop effective stewardship against foodborne diseases. In particular, veterinary practitioners should be aware of Campylobacteriosis outbreaks and alert caregivers of the possible pet–human bidirectional transmission of both *Campylobacter jejuni* and *Campylobacter upsaliensis*. Moreover, veterinarians should also advise pet parents of the possible risk of feeding their dog raw or undercooked protein sources and highlight the need for appropriate hygienic measures when petting a puppy or handling its feces.

The authors would also underline the possible role of such a case report in encouraging future studies about the prognostic indicators in Campylobacteriosis and the epidemiological factors facilitating the triage in hospitalized animals [43,44]. The availability of new technologies of investigation may contribute to a better understanding of zoonotic diseases spread and support prevention acts for public health [45]. 

## Figures and Tables

**Table 1:** Principal clinical findings and haemogas analysis results recorded during dog ER examination.

Clinical/Biochemical Findings	Results	Normality/ Reference Range [17]
Mental status	Lethargic, dull	Bright and alert
% of dehydration	5–8	Absent
Rectal temperature (°C)	40.1	38–39.2
Heart rate (bpm)	138	100–140
Respiratory rate (brpm)	22	12–20
Capillary refill time (s)	2	<2
Body condition score	3.5/9	5/9
pH	7.4	7.32–7.50 *
pCO^2^ (mmHg)	38	33–50 *
HCO^3−^ (mmol/L)	26	18–26 *
Base excess	2	−2 + 2 *

* Referred to blood collected by venipuncture.

**Table 2:** Main biochemical findings recorded during diagnostic workup.

Biochemical Findings	Results	Reference Interval
Hct (%)	55	42–58
WBC (10^3^/µL)	14.3	4.7–11.15
RBC (10^6^/µL)	8.5	6.13–8.52
Albumin (g/dL)	2.9	3–3.7
TP (g/dL)	6.6	5.7–7–1
BUN (mg/dL)	48	19–45
Crea (mg/dL)	0.8	0.76–1.24
Ca^2+^ (mg/dL)	8	9–11–5
Phos (mg/dL)	4	1.9–5.9
Na^+^ (mEq/L)	141	143–151
K^+^ (mEq/L)	4	3.9–4.9
Glucose (mg/dL)	92	85–123
ALT (IU/L)	121	10–94
ALP (IU/L)	220	10–250
Basal cortisol (µg/dL)	2.6	1.8–9

Alb: albumin; ALP: alkaline phosphatase; ALT: alaninoaminotransferase; BUN: blood urea nitrogen; CREA: creatinine; Hct: haematocrit; Phos: phosphorus; TP: total protein; RBC: red blood cells; WBC: white blood cells.

**Table 3:** Nutritional composition of the PRD, estimated by Myvetdiet© software. Data are provided on the total diet, as fed. Red text indicates nutrients whose intake is above or below minimum nutritional requirements according to [21,22].

Macronutrients		
Proteins: 41.61 g	Lipids: 28.15 g	Cholesterol: 150 mg
Carbohydrates: 12.98 g	Total Fiber: 1.23 g	Soluble Fiber: 0.31 g
Insoluble fiber: 0.37 g		
Minerals		
Potassium: 772.86 mg	Calcium: 22 mg	Sodium: 287.75 mg
Phosphorus: 445.50 mg	Zinc: 3.02 mg	Magnesium: 69.10 mg
Copper: 0.20 mg	Selenium: 26.90 µg	Chlorine: 220 mg
Iodine: 12 µg	Manganese: 0.23 mg	Iron: 2.43 mg
Vitamins		
Vitamin B1: 0.34 mg	Vitamin B2: 0.44 mg	Niacin: 12.94 mg
Niacin: 12.94 mg	Vitamin C: 8.50 mg	Vitamin B5: 2.35 mg
Vitamin B6: 0.68 mg	Total folates: 80.25 µg	Vitamin D: 0.20 µg
Biotin: 6 µg	Vitamin B12: 2 µg	Vitamin A eq.: 12.20 µg
ß-carotene eq.: 10,320 µg	Vitamin E: 4.76 mg	
Fatty acids profile		
Total sat. fatty acids: 5.59 g	Total mono fatty acids: 16.96 g	Total poly fatty acids: 3.52 g
Linoleic acid (w6): 2.76 g	Linolenic acid (w3): 0.28 g	Arachidonic acid (w6): 0.26 g
EPA (w3): 0.02 g	DHA (w3): 0.06 g	EPA + DHA: 0.08 g
Aminoacidic profile		
Tryptophan: 461.25 mg	Threonine: 1981.25 mg	Isoleucine: 2064.25 mg
Leucine: 3470 mg	Lysine: 3663.75 mg	Methionine: 1293.50 mg
Cystine: 546.25 mg	Phenylalanine: 1774.75 mg	Tyrosine: 1435.50 mg
Valina: 2192.50 mg	Arginine: 2786 mg	Histidine: 1437.50 mg
Taurine: nc		

nc: not calculated.

**Table 4:** Results of fecal culture, PCR assay, susceptibility testing and FISH of both the puppy and the owner (where deemed).

	Owner Fecal Culture	Puppy Fecal Culture	Puppy Colonic Biopsies Culture	Owner Fecal PCR Assay	Puppy Fecal PCR Assay	Puppy Susceptibility Testing	Puppy FISH on Colonic Biopsies
*C. jejuni*	x	n.i.	x	x	x	x	x
*C. upsaliensis*	x	x	n.i.	x	x	x	n.i.

**Table 5:** Results of fecal cultures, PCR assays, FISH of both the puppy and the owner (where deemed), antibiotic susceptibility testing (AST).

Bacterial Species	Owner Fecal Culture	Puppy Fecal Culture	Puppy Colonic Biopsies Culture	Puppy FISH Colonic Biopsies	Owner Fecal PCR	Puppy Fecal PCR	Puppy AST
*C. jejuni*	+	n.i.	+	+	+	+	S: NAL (8 mg/mL), CIP (<0.12 mg/mL), GEN (1 mg/mL), ERY (≤1 mg/mL), STR (4 mg/mL) R: TET (64 mg/mL)
*C. upsaliensis*	+	+	n.i.	n.i.	+	+	NA: CIP (<2 mg/mL), ERY (<0.016 mg/mL), STR (<0.064 mg/mL), TET (<0.016 mg/mL)

C.: Campylobacter; +: positive identification; n.i.: non-isolated or identified; S: Sensitive; I: Intermediate; R: Resistant; NA: not applicable; NAL: Nalidixic Acid; STR: Streptomycin; ERY: Erytromycin; TET: tetracycline; CIP: ciprofloxacin [26].

**Table 6:** Major findings described by the pathologist (L.A.) while assessing biopsies of the puppy duodenal and colonic mucosa. Description has been carried out in accordance with WSAVA Gastrointestinal Standardization Group guidelines [27].

	Morphological Features			
	Normal	Mild	Moderate	Marked
Surface epithelial injury/Villous stunting	LG			S
Crypt hyperplasia/Crypt distension *	S	L		
Crypt dilation/distortion *	LS			
Fibrosis/atrophy	LG	S		
	Inflammation			
	Normal	Mild	Moderate	Marked
Intra-epitelial lymphocytes ^§^	G		S	
Lamina propria Lymphocytes and plasma cells		G	L	S
Lamina propria eosinophils	LSG			
Lamina propria neutrophils	LSG			
Lamina propria macrophages	LSG			
	Other notes			
Edema	LSG			
Superficial erosions	L			
	Final diagnosis			
	Mild chronic gastritis; Moderate-to-severe lymphoplasmacellular enteritis with signs of fibrosis; Moderate lymphoplasmacellular colitis with signs of erosions.			

L: Large bowel biopsies (colon and ileum); S: Small bowel biopsies (duodenum e jejunum); G: gastric biopsies. * Non-applicable for G; ^§^ non applicable for L.

## Data Availability

The datasets used or analyzed during the current study are available from the corresponding author upon reasonable request.

## References

[1] García-Fernández A., Dionisi A.M., Arena S., Iglesias-Torrens Y., Carattoli A., Luzzi I. (2018). Human Campylobacteriosis in Italy: Emergence of Multi-Drug Resistance to Ciprofloxacin, Tetracycline, and Erythromycin. Front. Microbiol..

[2] European Food Safety Authority, European Centre for Disease Prevention and Control (2017). The European Union Summary Report on Trends and Sources of Zoonoses, Zoonotic Agents and Food-borne Outbreaks in 2016. EFSA J..

[3] European Food Safety Authority, European Centre for Disease Prevention and Control (2019). The European Union Summary Report on Trends and Sources of Zoonoses, Zoonotic Agents and Food-borne Outbreaks in 2018. EFSA J..

[4] European Food Safety Authority, European Centre for Disease Prevention and Control (2021). The European Union One Health 2019 Zoonoses Report. EFSA J..

[5] Fredriksson-Ahomaa M., Heikkilä T., Pernu N., Kovanen S., Hielm-Björkman A., Kivistö R. (2017). Raw Meat-Based Diets in Dogs and Cats. Vet. Sci..

[6] Vinassa M., Vergnano D., Valle E., Giribaldi M., Nery J., Prola L., Bergero D., Schiavone A. (2020). Profiling Italian Cat and Dog Owners’ Perceptions of Pet Food Quality Traits. BMC Vet. Res..

[7] Hoummady S., Fantinati M., Maso D., Bynens A., Banuls D., Santos N.R., Roche M., Priymenko N. (2022). Comparison of Canine Owner Profile According to Food Choice: An Online Preliminary Survey in France. BMC Vet. Res..

[8] Empert-Gallegos A., Hill S., Yam P.S. (2020). Insights into Dog Owner Perspectives on Risks, Benefits, and Nutritional Value of Raw Diets Compared to Commercial Cooked Diets. PeerJ.

[9] Morelli G., Bastianello S., Catellani P., Ricci R. (2019). Raw Meat-Based Diets for Dogs: Survey of Owners’ Motivations, Attitudes and Practices. BMC Vet. Res..

[10] Freeman L.M., Chandler M.L., Hamper B.A., Weeth L.P. (2013). Current Knowledge about the Risks and Benefits of Raw Meat–Based Diets for Dogs and Cats. J. Am. Vet. Med. Assoc..

[11] APPA (2018). The 2017–2018 APPA National Pet Owners Survey Debut.

[12] European Food Safety Authority, European Centre for Disease Prevention and Control (2018). The European Union Summary Report on Trends and Sources of Zoonoses, Zoonotic Agents and Food-borne Outbreaks in 2017. EFSA J..

[13] Gras L.M., Smid J.H., Wagenaar J.A., Koene M.G.J., Havelaar A.H., Friesema I.H.M., French N.P., Flemming C., Galson J.D., Graziani C. (2013). Increased Risk for *Campylobacter jejuni* and *C. coli* Infection of Pet Origin in Dog Owners and Evidence for Genetic Association between Strains Causing Infection in Humans and Their Pets. Epidemiol. Infect..

[14] Davies R.H., Lawes J.R., Wales A.D. (2019). Raw Diets for Dogs and Cats: A Review, with Particular Reference to Microbiological Hazards. J. Small Anim. Pract..

[15] Ahmed F., Cappai M.G., Morrone S., Cavallo L., Berlinguer F., Dessì G., Tamponi C., Scala A., Varcasia A. (2021). Raw meat based diet (RMBD) for household pets as potential door opener to parasitic load of domestic and urban environment. Revival of understated zoonotic hazards? A review. One Health.

[16] Waltham Waltham Faeces Scoring System. https://waltham.com/media/2020-05/waltham-scoring.pdf?VersionId=27x4BJc1CDcBNfM04N6jLXGJUCEw.vc_.

[17] Viganò F. (2002). Capire l’emogasanalisi:Un Metodo Semplice per Interpretare i più Comuni Disturbi Acido-Base. Veterinaria.

[18] Candellone A., Girolami F., Badino P., Jarriyawattanachaikul W., Odore R. (2022). Changes in the Oxidative Stress Status of Dogs Affected by Acute Enteropathies. Vet. Sci..

[19] Neumann S., Steingräber L., Herold L. (2022). Investigation of Procalcitonin and Beta-defensin2 in the Serum and Feces of Dogs with Acute Diarrhea. Vet. Clin. Pathol..

[20] Bovens C., Tennant K., Reeve J., Murphy K.F. (2014). Basal Serum Cortisol Concentration as a Screening Test for Hypoadrenocorticism in Dogs. J. Vet. Intern. Med..

[21] National Research Council (2006). Nutrient Requirements of Dogs and Cats.

[22] FEDAF FEDIAF Nutritional Guidelines for Complete and Complementary Pet Food for Cats and Dogs. https://europeanpetfood.org/wp-content/uploads/2022/03/Updated-Nutritional-Guidelines.pdf.

[23] Weese J.S. (2011). Bacterial Enteritis in Dogs and Cats: Diagnosis, Therapy, and Zoonotic Potential. Vet. Clin. N. Am. Small Anim. Pract..

[24] Nüesch-Inderbinen M., Treier A., Zurfluh K., Stephan R. (2019). Raw Meat-Based Diets for Companion Animals: A Potential Source of Transmission of Pathogenic and Antimicrobial-Resistant Enterobacteriaceae. R. Soc. Open Sci..

[25] (2019). Microbiology of the Food Chain—Horizontal Method for Detection and Enumeration of *Campylobacter* spp.—Part 1: Detection Method.

[26] Aerts M., Battisti A., Hendriksen R., Kempf I., Teale C., Tenhagen B., Veldman K., Wasyl D., Guerra B., Liébana E. (2019). Technical Specifications on Harmonised Monitoring of Antimicrobial Resistance in Zoonotic and Indicator Bacteria from Food-producing Animals and Food. EFSA J..

[27] WSAVA Gastrointestinal Standardization Group (2008). Summary of Findings and Reports of the WSAVA Gastrointestinal Standardization Group.

[28] Humphrey T.J., Martin K.W., Slader J., Durham K. (2001). *Campylobacter* spp. in the Kitchen: Spread and Persistence. J. Appl. Microbiol..

[29] Selwet M., Cłapa T., Galbas M., Słomski R., Porzucek F. (2015). The Prevalence of *Campylobacter* spp. and Occurrence of Virulence Genes Isolated from Dogs. Pol. J. Microbiol..

[30] Murawska M., Sypecka M., Bartosik J., Kwiecień E., Rzewuska M., Sałamaszyńska-Guz A. (2022). Should We Consider Them as a Threat? Antimicrobial Resistance, Virulence Potential and Genetic Diversity of *Campylobacter* spp. Isolated from Varsovian Dogs. Antibiotics.

[31] Selwet M., Galbas M., Słomski R., Cłapa T., Porzucek F. (2016). Monitoring of Virulence Genes, Drug-Resistance in *Campylobacter coli* Isolated from Golden Retrievers. Pol. J. Microbiol..

[32] Andrzejewska M., Klawe J.J., Szczepańska B., Spica D. (2011). Occurrence of Virulence Genes among *Campylobacter jejuni* and *Campylobacter coli* Isolates from Domestic Animals and Children. Pol. J. Vet. Sci..

[33] Westgarth C., Pinchbeck G.L., Bradshaw J.W.S., Dawson S., Gaskell R.M., Christley R.M. (2008). Dog-Human and Dog-Dog Interactions of 260 Dog-Owning Households in a Community in Cheshire. Vet. Rec..

[34] Solís D., Toro M., Navarrete P., Faúndez P., Reyes-Jara A. (2022). Microbiological Quality and Presence of Foodborne Pathogens in Raw and Extruded Canine Diets and Canine Fecal Samples. Front. Vet. Sci..

[35] Candellone A., Cerquetella M., Girolami F., Badino P., Odore R. (2020). Acute Diarrhea in Dogs: Current Management and Potential Role of Dietary Polyphenols Supplementation. Antioxidants.

[36] Rendle D.I., Page S.W. (2018). Antimicrobial Resistance in Companion Animals. Equine Vet. J..

[37] World Health Organization (WHO) (1983). Antimicrobial Resistance.

[38] Palma E., Tilocca B., Roncada P. (2020). Antimicrobial Resistance in Veterinary Medicine: An Overview. Int. J. Mol. Sci..

[39] Pomba C., Rantala M., Greko C., Baptiste K.E., Catry B., van Duijkeren E., Mateus A., Moreno M.A., Pyörälä S., Ružauskas M. (2017). Public Health Risk of Antimicrobial Resistance Transfer from Companion Animals. J. Antimicrob. Chemother..

[40] Ge B., Wang F., Sjölund-Karlsson M., McDermott P.F. (2013). Antimicrobial Resistance in *Campylobacter*: Susceptibility Testing Methods and Resistance Trends. J. Microbiol. Methods.

[41] Iannino F., Salucci S., di Donato G., Badagliacca P., Vincifori G., di Giannatale E. (2019). *Campylobacter* and Antimicrobial Resistance in Dogs and Humans: “One Health” in Practice. Vet. Ital..

[42] Kaakoush N.O., Castaño-Rodríguez N., Mitchell H.M., Man S.M. (2015). Global Epidemiology of *Campylobacter* Infection. Clin. Microbiol. Rev..

[43] Chalifoux N.V., Parker S.E., Cosford K.L. (2021). Prognostic Indicators at Presentation for Canine Parvoviral Enteritis: 322 Cases (2001–2018). J. Vet. Emerg. Crit. Care.

[44] Machado I.C., Nunes T., Maximino M., Malato J., Tavares L., Almeida V., Sepúlveda N., Gil S. (2023). Epidemiologic Factors Supporting Triage of Infected Dog Patients Admitted to a Veterinary Hospital Biological Isolation and Containment Unit. Vet. Sci..

[45] Carella E., Orusa T., Viani A., Meloni D., Borgogno-Mondino E., Orusa R. (2022). An Integrated, Tentative Remote-Sensing Approach Based on NDVI Entropy to Model Canine Distemper Virus in Wildlife and to Prompt Science-Based Management Policies. Animals.

